# Acute phase proteins and markers of oxidative stress to assess the severity of the pulmonary hypertension in heartworm-infected dogs

**DOI:** 10.1186/s13071-017-2426-8

**Published:** 2017-11-09

**Authors:** Elena Carretón, José Joaquín Cerón, Silvia Martínez-Subiela, Asta Tvarijonaviciute, Alicia Caro-Vadillo, José Alberto Montoya-Alonso

**Affiliations:** 1 Faculty of Veterinary Medicine, Research Institute of Biomedical and Health Sciences (IUIBS), University of Las Palmas de Gran Canaria, Trasmontaña s/n, 35413-Arucas, Las Palmas, Gran Canaria Spain; 2 Interdisciplinary Laboratory of Clinical Analysis (Interlab-UMU), Veterinary School, University of Murcia, Murcia, Spain; 3 Medicina y Cirugía Animal, Faculty of Veterinary Medicine, University Complutense of Madrid, Madrid, Spain

**Keywords:** Heartworm, *Dirofilaria immitis*, Pulmonary hypertension, Pulmonary artery, Endarteritis, Acute phase proteins, Inflammation, Oxidative stress, Canine

## Abstract

**Background:**

Canine heartworm infection is characterized by pulmonary endarteritis and pulmonary hypertension (PH). The aim of the present study was to evaluate the relationship between PH with the concentrations of different positive (C-reactive protein [CRP] and haptoglobin [Hp]) and negative (albumin and paraoxonase-1 [PON-1]) acute phase proteins (APP), as well as the oxidative stress, by measuring glutathione peroxidase (GPx) and total antioxidant capacity (TAC) in 27 heartworm-infected dogs on Day 0 (diagnosis) and Day 120 (1 month after the last adulticide injection). Presence/absence of PH was determined by the Right Pulmonary Artery Distensibility (RPAD) Index.

**Results:**

On Day 0, 62.9% of the dogs showed PH. Concentrations of CRP and Hp were higher in dogs with PH, especially in dogs with moderate-severe PH (*P* < 0.005 and *P* < 0.05, respectively). Albumin and PON-1 concentrations were higher in dogs without PH (*P* < 0.05 for albumin). On Day 120, 59.2% of the dogs presented with PH; CRP decreased while Hp increased (*P* < 0.005 and *P* < 0.05, respectively). Also, albumin and PON-1 rose, especially in absence of PH. There were not significant changes in the serum values of GPx and TAC.

**Conclusions:**

CRP and Hp have a potential prognostic role in dogs with dirofilariasis because increases in positive APP correlated with presence and severity of PH. CRP decreased, but Hp persisted at an elevated level in dogs with PH 1 month after the end of the adulticide treatment. CRP and Hp could work as early biomarkers of PH and be useful to stage the disease and to monitor the evolution of the patient and indirectly evaluate the persistence of arterial damage after the parasites have been eliminated. Albumin and PON-1 also showed potential value as markers of PH, although further research is necessary to determine its utility.

## Background

Canine heartworm disease is a chronic disease caused by *Dirofilaria immitis* and is characterized by the presence of the adult parasites in the pulmonary arteries. The worms cause proliferative endarteritis from the first days after they reach the caudal pulmonary arteries. This damage causes chronic vascular remodeling that leads to the development of pulmonary hypertension (PH), which can induce right-sided congestive heart failure [1, 2]. This pathogenic reaction is caused by the host’s response to the worm as well as the bacterial endosymbiont *Wolbachia pipientis*, which triggers the release of proinflammatory and chemotactic cytokines, inducing cellular infiltration and amplification of the inflammatory response [3, 4].

Transthoracic Doppler echocardiography is the method of choice to diagnose naturally occurring PH in veterinary patients [5]. But when tricuspid regurgitation and/or pulmonary insufficiency are not present, the diagnosis of PH is based on indirect and subjective parameters, which only help partially to quantify the disease severity. Recently, the Right Pulmonary Artery Distensibility Index (RPAD Index), which is calculated as the difference in diameter of the right pulmonary artery in systole and diastole as measured by M-mode, was validated as a valuable method to estimate the presence and severity of PH in heartworm-infected dogs [6].

Acute phase proteins (APP) are proteins present in the plasma whose concentrations modify in response to trauma, infection or inflammation. APP are considered part of the innate host defense system, and its quantification provides valuable clinical information in the diagnosis, monitoring and prognosis of various diseases [7, 8]. The APP are classified into two groups based on their response to the triggering event: negative APP (eg, albumin and paraoxonase-1 [PON-1]) are those whose levels are diminished and positive APP (eg, C-reactive protein [CRP] and haptoglobin [Hp]) are those whose levels are increased when there is an acute phase response [9–11].

Previous studies have reported an acute phase response in dogs with *D. immitis*, characterized by variations of APP with increases in CRP, decreases in albumin and PON-1 and a divergence in the behavior between CRP and Hp [12]. Furthermore, a study monitored the progression of the disease through the determination of the APP after pretreatment with doxycycline and ivermectin and after the adulticide treatment with melarsomine by showing an improvement in the concentrations of APP as the pathology disappeared [13].

Activity of antioxidant enzymes, such as glutathione peroxidase (GPx), has been quantified in plasma as measures of antioxidant capabilities. Studies found that GPx deficiency was associated with endothelial dysfunction and vascular structural abnormalities, including increased matrix deposition and intimal thickening [14, 15]. Some results suggest that GPx plays a critical role in regulating proinflammatory pathways; failing to regulate these proinflammatory pathways facilitates an inflammatory and activated endothelium leading to endothelial dysfunction [16]. Total antioxidant capacity (TAC) is the measure of the amount of free radicals scavenged by a test solution. It is an analyte frequently used to assess the antioxidant status of biological samples and can evaluate the antioxidant response against the free radicals produced in a given disease [17].

A novel study found a correlation between CRP concentrations with severity of pulmonary arterial damage and PH in dogs with *D. immitis*, finding significantly increased CRP in animals with mild or severe PH [18]. This fact has made it possible to believe that CRP could be used for staging and monitoring the disease in dogs and may work as an early biomarker of PH. However, there are no studies on other APP or markers of oxidative stress. Therefore, the aim of the present study was to evaluate the relationship between PH with the concentrations of different positive (C-reactive protein [CRP] and haptoglobin [Hp]) and negative (albumin and paraoxonase-1 [PON-1]) APP, as well as the oxidative stress by measuring GPx and total antioxidant capacity (TAC) in heartworm-infected dogs at the beginning and the end of the adulticide treatment to evaluate their utility as diagnostic markers of PH.

## Methods

In the present study, 27 heartworm-infected dogs positive to circulating antigens were included. The dogs lived in Gran Canaria (Spain), a hyperendemic area of *D. immitis* [19, 20]. None of the animals had received previous treatment for heartworm infection. A complete record was kept for each animal, including identification (age, sex and breed), clinical history and demographic data. There were 12 females and 15 males; the mean age was 4.7 years (from 3 to 9 years). The dogs were divided into two groups (<4 years, *n* = 13, and >4 years, *n* = 14) when the age was evaluated. By breed, 13 were mongrel dogs and 14 were purebreds (13 different breeds). All were positive to circulating *D. immitis* antigens using a commercial immunochromatographic test kit (Uranotest Dirofilaria®, Urano Vet SL, Barcelona, Spain). Dogs were further evaluated for the presence or absence of microfilariae using a modified Knott’s test. In addition, a group of 10 healthy dogs (6 males and 4 females), with ages between 2 and 8 years, of different breeds were used as control group. All these dogs showed no abnormality on physical examination and on routine hematologic and biochemical analyses and were negative for circulating *D. immitis* antigens.

On the day of the diagnosis (Day 0) and at the end of the treatment (Day 120), dogs underwent echocardiographic examination using an ultrasound machine with spectral and color Doppler and multifrequency probes (5.5–10 MHz) (Logic P5, General Electric, New York, USA). Presence or absence of PH was determined by the Right Pulmonary Artery Distensibility Index (RPAD Index) as previously described [6]. The parasite burden visible in the pulmonary arteries and right cardiac chambers was also assessed by echocardiography.

Blood was drawn from the cephalic vein of each animal on Day 0 and Day 120 to measure APP and markers of oxidative stress. Blood was collected into tubes without anticoagulant and serum was obtained and frozen (−20 °C) until analysis. Concentrations of CRP were measured using a human immunoturbidimetric test (CRP OSR 6147 Olympus Life and Material Science Europe GmbH, Lismeehan, O’Callaghan’s Mills, Co. Clare, Ireland); Hp concentrations were measured using a colorimetric method (kit haptoglobin TrideltaPhase range SAA kit, Tridelta Development Ltd., Co. Kildare, Ireland); albumin was determined using a Bromocresol green reagent [Albumin OSR 6102 Olympus Life and Material Science Europe GmbH Lismeehan, O’Callaghan’s Mills, Co. Clare, Ireland]; and serum PON-1 activity was determined using p-nitrophenyl acetate as substrate. All techniques had previously been validated for use in dogs [21–24]. GPx and TAC were analyzed as previously described [25, 26]. All determinations were performed in serum on an automated biochemistry analyzer (Olympus AU600 Automatic Chemistry Analyzer, Olympus Europe GmbH, Hamburg, Germany).

The infected dogs underwent adulticide treatment following the American Heartworm Society recommended management protocol [27]. In short, on Day 0 the dog was diagnosed as heartworm positive, starting with the administration of doxycycline (10 mg/kg BID) for 4 weeks and the monthly heartworm preventive based on ivermectin (6 μg/kg). On Day 60 the dog was treated with the first intramuscular injection of melarsomine (2.5 mg/kg), followed on Day 90 by a second injection and a third injection on Day 91. On Day 120, the dog was tested for presence of microfilariae and discharged. On Day 271, 6 months after completion, the adulticide efficacy was confirmed with an antigen test. Exercise restriction was recommended until at least 1 month after the last melarsomine injection.

The data were analyzed using the SPSS Base 22.0 software for Windows. The nonparametrical Mann-Whitney U test was used to compare results obtained for dogs negative and positive to heartworm disease and also for the comparison between diseased dogs with PH and without PH. Kruskal-Wallis with Dunn’s multiple comparison test was used for the comparison between dogs grouped by PH status. Wilcoxon signed rank test was used to compare the results obtained for the different analytes at two different times (Day 0 and Day 120). In all cases, a *P* value <0.05 was determined as significant.

All of the owners gave their consent to participate in this study. The study was approved by the ethical committee of the Veterinary Medicine Service of the University of Las Palmas de Gran Canaria and was carried out in accordance with the current European legislation on animal protection.

## Results

Absence or presence of PH, as well as PH severity, was based on the determination of the RPAD Index. An Index between 35 and 28% was correlated with mild PH (30–55 mmHg), between 27 and 23% with moderate PH (56–79 mmHg), and 22% or less with severe PH (>79 mmHg). On Day 0, 62.9% (17/27) of the dogs showed PH (25.9% mild, 37% moderate-severe PH). Although the RPAD Indexes were lower in dogs <4 years compared with older dogs (>4 years), the differences were not significant.

No worms were visualized by echocardiography in any of the studied dogs at the end of the treatment (Day 120) and 59.2% (16/27) presented PH (37 mild, 22.2% moderate-severe PH). PH was still present in 70.6% (12/17) of the dogs with PH at the beginning of the treatment, and 40% (4/10) of the dogs with normal pulmonary pressure at the beginning of the treatment showed PH on Day 120.

**Fig. 1:**
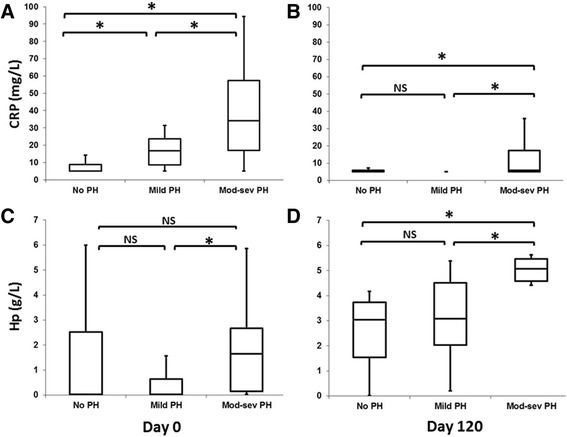
Serum concentrations of positive acute phase proteins CRP on Day 0 (**a**) and Day 120 (**b**), and Hp on Day 0 (**c**) and Day 120 (**d**), in dogs with heartworm without pulmonary hypertension (no HP) (RPAD Index ≥36%), with mild pulmonary hypertension (mild HP) (RPAD Index 28–35%) and moderate or severe pulmonary hypertension (Mod-sev HP) (RPAD Index ≤27%). The box plots represent median (solid horizontal lines within boxes), 25th and 75th percentiles (boxes) and minimum and maximum values (whiskers). (*): significant differences between groups. NS: not significant

At the beginning of the study, the concentrations of CRP in the infected dogs (mean: 23.4 mg/L) were significantly higher compared with those obtained in the control group (mean: 6.45; SD: 2.36; *P* < 0.05). By age, CRP was significantly higher in older dogs (mean: 32.1 mg/L versus 13.9 mg/L; *P* < 0.005). CRP concentrations were higher in dogs with PH (mean: 30.2 mg/L) when comparing with dogs that had normal pulmonary pressure (mean: 12.03 mg/L; *P* < 0.005). CRP concentrations were higher in dogs with moderate-severe PH (mean: 39.6 mg/L) when compared with dogs that had mild PH (mean: 16.8 mg/L; *P* < 0.005) (Fig. 1).

On Day 120, CRP concentrations decreased in all animals (mean: 7.7 mg/L) as compared with Day 0 (*P* < 0.005). By age, means were 8.6 mg/L (<4 years) and 7.2 mg/L (>4 years), with no significant differences. By PH status, the results showed that CRP values were lower in dogs without PH (reaching a mean concentration of 5.6 mg/L) than in dogs with PH (mean: 9 mg/L; *P* < 0.05). By severity, the dogs with mild PH (mean: 6.5 mg/L) showed lower CRP values than those with moderate-severe PH (mean: 14 mg/L; *P* < 0.05) (see Fig. 1).

Concentrations of Hp were significantly decreased in dogs with heartworm on Day 0 (mean: 1.38 g/L) when compared with values obtained in the control group (mean: 2.16; SD: 0.86; *P* < 0.05). When age was evaluated, older dogs showed higher levels (mean: 1.9 g/L versus mean: 0.85 g/L), with no significant differences. When the RPAD Index was evaluated, concentrations were higher in dogs with PH (mean: 1.39 g/L) compared with dogs with normal pulmonary pressure (mean: 1.05 g/L), although the differences were not statistically significant. By severity, Hp concentrations were higher in dogs with moderate-severe PH (mean: 1.85 g/L) than in dogs with mild PH (mean: 0.86 g/L; *P* < 0.005) (see Fig. 1).

On Day 120, Hp concentrations increased in all animals (mean: 3.26 g/L; *P* < 0.05). Hp concentration was higher in older dogs (mean: 3.4 g/L versus mean: 2.9 g/L), with no significant differences; and it was higher in dogs with PH (mean: 3.68 g/L; *P* < 0.005). In addition, dogs with moderate-severe PH showed higher values at the end of the treatment than those with mild PH (mean: 5.03 g/L versus mean: 3.01 g/L; *P* < 0.005) (see Fig. 1).

**Fig. 2:**
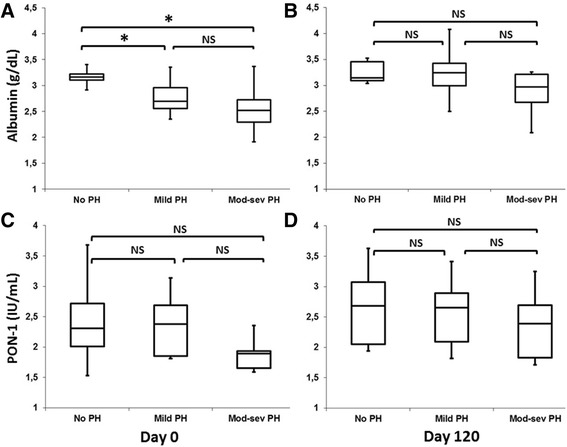
Serum concentrations of negative acute phase proteins albumin on Day 0 (**a**) and Day 120 (**b**), and PON-1 on Day 0 (**c**) and Day 120 (**d**) in dogs with heartworm without pulmonary hypertension (no HP) (RPAD Index ≥36%), with mild pulmonary hypertension (mild HP) (RPAD Index 28–35%) and moderate or severe pulmonary hypertension (Mod-sev HP) (RPAD Index ≤27%). The box plots represent median (solid vertical lines within boxes), 25th and 75th percentiles (boxes) and minimum and maximum values (whiskers). (*): significant differences between groups. NS: not significant

When negative APP were evaluated, it was found that concentrations on Day 0 were significantly diminished in dogs with heartworm (means: 2.8 g/dL, *P* < 0.005 for albumin and 2.24 IU/mL, *P* < 0.05 for PON-1) with regard to those obtained in dogs of the control group (mean 3.32, SD: 0.31 for albumin and mean: 2.79, SD: 0.55 for PON-1). Concentrations were lower in dogs with PH (means: 2.6 g/dL for albumin and 2.13 IU/mL for PON-1) when compared with dogs without PH (means: 3.1 for albumin g/dL and 2.45 IU/mL for PON-1) (*P* < 0.05 for albumin). When the severity of the PH was assessed, it was observed that negative APP concentrations were lower in dogs with moderate-severe PH (means: 2.5 g/dL for albumin and 1.98 IU/mL for PON-1) than in dogs with mild PH (means: 2.7 g/dL for albumin and 2.25 IU/mL for PON-1), the differences not being statistically significant (Fig. 2). No significant differences were observed between groups of age in negative APP.

On Day 120, albumin and PON-1 showed a significant increase when compared with Day 0 (*P* < 0.05 for albumin and PON-1). However, no significant differences were observed between groups with regard to the age or the presence or absence of PH.

Regarding the biomarkers of oxidative stress, GPx and TAC, there are not statistically significant differences in the serum values between dogs with and without heartworm disease. In addition, no significant changes were found between dogs before and after treatment and with and without hypertension.

## Discussion

The changes found in the group of heartworm-infected dogs for the positive (CRP and Hp) and negative APP (albumin and PON-1) on Day 0 compared with the healthy dogs probably reflect the inflammatory process associated with the disease, as previously described [12]. Furthermore, mean values of all analytes except Hp after treatment were below the upper limit of the reference range of our laboratory, which is 12 mg/L in the case of CRP, 3 g/L for Hp, 36 g/L for albumin and 4.3 IU/mL for PON-1. Therefore the study demonstrated normalization in most of the concentrations of the studied APP after the elimination of the parasites, similar to other research [12, 13], while the increases observed in Hp on Day 120 are similar to a previous study [13].

When the APP were assessed according to the PH status, it was observed that dogs with PH at the beginning of the treatment showed higher CRP concentrations; furthermore, these CRP concentrations increased with the severity, the highest being found in dogs with moderate-severe PH. Venco et al. [18] described similar findings, reporting that dogs with mild or severe PH presented significantly increased CRP. The behavior of the Hp was similar; Hp increased in dogs with PH, the greater increase being in those with moderate-severe PH.

PH is a chronic condition caused by the presence of heartworms, mainly through the development of proliferative endarteritis and fibrosis at the capillary beds, among other factors [1, 2, 28]. It is possible that CRP play an important role in the chronic process of remodeling of the vasculature and its concentrations correlate with the severity and chronicity of the disease [10, 18]. Studies have been published that show that CRP is able to enhance smooth muscle proliferation and secretion of endothelin-1, a potent vasoconstrictor in humans [29], and mild CRP elevations have been reported from clinically early stages of heartworm infection [30]. Because PH is a frequent and serious complication in heartworm disease, its objective determination is important. The detection of increased concentrations of CRP and Hp may be used to determine a high level of suspicion of pulmonary hypertension in the animals affected, so these APP may be useful as early biomarkers of PH and to stage the disease.

After the adulticide treatment, CRP was lower in the absence of PH compared with dogs with persistent PH, especially in those with moderate-severe PH. Regarding Hp, concentrations were higher in dogs with moderate-severe PH. At the end of the treatment, 70.6% of the dogs with PH on Day 0 still presented this pathological alteration, which indicates the persistence of the vascular damage 1 month after the end of the adulticide treatment and when the parasites are most likely to be dead. Furthermore, 40% of the dogs without PH developed PH during the treatment. This may be caused by either embolism of dead heartworms, which can cause acute clinical signs of PH, or by proliferative intimal changes, which lead to structural damage to the vasculature and sustained PH [31, 32]. However, less severe arterial lesions and virtual absence of emboli in the currently recommended adulticide protocol are observed [30], and further research should be done to assess the degree of reversibility of vascular damage in dogs under this protocol.

Although it is not possible to determine the duration of the infection – and, therefore, the chronicity – in the naturally infected dogs from this study, consideration must be given to this fact. It could be said that older dogs are more commonly affected by chronic infections. The CRP was significantly higher in older dogs on Day 0, which could reflect the chronicity of the infection. The heterogeneity of animals makes it impossible to discuss the possible influence of the breed in the results.

These data highlight the need to keep monitoring the dogs after finishing the adulticide treatment and assess the evolution of PH. For this, CRP and Hp could be useful to monitor the evolution of the patient and indirectly evaluate the persistence of arterial damage once the parasites have been eliminated. Also, increases of CRP after the treatment might spark a suspicion of development of PH or worsening in these patients.

The negative APP (albumin and PON-1) behaved similarly to previous studies and decreased in dogs with heartworm [12]. Also, they showed an association between presence and absence of PH as well as severity of PH, since the dogs with moderate-severe PH showed lower values. However, this association was not statistically significant, and further research must be carried out to assess their clinical utility.

No differences were found in the markers of oxidative stress between heartworm-positive and control groups, which may indicate that no important phenomena of oxidative damage were occurring in heartworm-infected dogs or maybe that the assays used for oxidative damage evaluation were not sensitive enough to detect changes in this disease. Previous studies showed a relationship between deficiency in markers of oxidative stress and endothelial dysfunction [14–16]. Endothelial dysfunction is a pathological condition mainly defined by an imbalance between substances and is characterized by proinflammatory, vasoconstricting, prothrombotic and proliferative states [33, 34]. As proliferative endarteritis is represented by these alterations, a relationship between the markers of oxidative stress GPx and TAC was sought. As this is the first research to evaluate the markers of oxidative stress in heartworm disease, further studies if possible using other biomarkers for the assessment of stress responses are recommended.

## Conclusions

The results showed that positive APP, CRP and Hp increase in dogs with PH. Although further research is necessary, CRP and Hp have a potential prognostic role in dogs with dirofilariasis as increases in positive APP correlated with presence and severity of PH. Moreover, high concentrations of these APP persisted in dogs with PH 1 month after the end of the adulticide treatment, while in the case of CRP they returned to normal in dogs in the absence of PH; therefore, CRP could indirectly evaluate the persistence of arterial damage once the parasites have been eliminated.

The negative APP also showed a potential value as markers of PH, although further research is necessary to determine the utility.

## Abbreviations

APP: Acute phase proteins
CRP: C-reactive protein
GPx: Glutathione peroxidase
Hp: Haptoglobin
PH: Pulmonary hypertension
PON-1: Paraoxonase-1
RPAD Index: Right pulmonary artery distensibility index
TAC: Total antioxidant capacity
